# Community-Acquired *Clostridium difficile* Infection, Queensland, Australia

**DOI:** 10.3201/eid2209.151115

**Published:** 2016-09

**Authors:** Luis Furuya-Kanamori, Laith Yakob, Thomas V. Riley, David L. Paterson, Peter Baker, Samantha J. McKenzie, Jenny Robson, Archie C.A. Clements

**Affiliations:** The Australian National University, Canberra, Australian Capital Territory, Australia (L. Furuya-Kanamori, A.C.A. Clements);; London School of Hygiene and Tropical Medicine, London, UK (L. Yakob);; The University of Western Australia and PathWest Laboratory Medicine, Nedlands, Western Australia, Australia (T.V. Riley);; The University of Queensland, Herston, Queensland, Australia (D.L. Paterson, P. Baker);; The University of Queensland, St. Lucia, Queensland, Australia (S.L. McKenzie);; Sullivan Nicolaides Pathology, Taringa, Queensland, Australia (J. Robson)

**Keywords:** Clostridium difficile, community-acquired infections, medication, exposure, Australia, bacteria, enteric infections

**To the Editor:** In Queensland, Australia, a steady increase in community-acquired (CA) *Clostridium difficile* infections (CDI) during 2003–2012 could not be explained by patients’ demographic characteristics or environmental factors (*1*). Several risk factors have been implicated in the increased rates of CA-CDI, primarily exposure to antimicrobial drugs, gastric acid–suppression drugs, and corticosteroids (*2*). Given the recent rise in prescription of corticosteroids and proton pump inhibitors in Australia, we hypothesized that the observed increase in CA-CDI was associated with increased drug prescriptions.

To test our hypothesis, we analyzed a subset of data used in a previous study (*1*), which included fecal samples from patients seen by general practitioners in the community from January 2008 through December 2012. The samples were submitted to Sullivan Nicolaides Pathology (Taringa, Queensland, Australia) for *C. difficile* toxin gene detection. After samples submitted from healthcare facilities and nursing homes were excluded, the final dataset contained data from 14,330 fecal samples. We aggregated the data by patient sex, age categories, year, and statistical area level 4 (SA4). For each sex-age-year-SA4 group, we used as numerators the numbers of CA-CDI cases identified and as denominators the numbers of samples submitted for microbiological testing. 

The Australian Department of Human Services provided data from the Pharmaceutical Benefits Scheme. The quantities of 11 anatomic therapeutic chemical drugs were accessed by patient sex, age group, year, and SA4. Corresponding with the CA-CDI data, medication data to be analyzed were then aggregated by sex, age group, year, and SA4.

For each medication, we built binomial logistic regression models, using CA-CDI status as the outcome, in a Bayesian framework, incorporating fixed effects for sex, age group, quantity of drug prescribed, year (2008–2012), and spatially unstructured random effects at the SA4 level. After performing an initial burn-in, we stored and summarized 1,000 values from the posterior distribution of each parameter by using descriptive statistics (posterior mean, 95% posterior credible interval [95% CrI], and p value). We examined multiple pairwise comparisons of CA-CDI and medication exposure; thus, we used the Holm adjustment for p values to avoid α inflation and to control the familywise error rate.

Of the 14,330 fecal samples tested, 1,430 (10%) were positive for *C. difficile*. The proportion of positive fecal samples increased over the 5-year period, from 7.10% in 2008 to 12.72% in 2011 and 11.48% in 2012 (p<0.001). After adjusting the regression models for sex, age group, temporal pattern, and spatial distribution, we found that exposure to antimycobacterial drugs (odds ratio [OR] 1.09; 95% CrI 1.02–1.16) and anthelmintic drugs (OR 1.07; 95% CrI 1.01–1.13) were associated with increased odds of CA-CDI. After post hoc Holm adjustments, no statistically significant association between medication exposure and CA-CDI was observed (Table).

**Table:** Binomial logistic regression models for medication exposure adjusted for sex, age group, temporal pattern, and spatial distribution among patients with community-acquired *Clostridium difficile* infection, Queensland, Australia, 2003–2012

Medication exposure	Odds ratio (95% credible interval)	p value	Holm-adjusted p value
Drugs for acid-related disorders	1.052 (0.943–1.163)	0.348	0.819
Drugs for constipation	1.056 (0.963–1.151)	0.235	0.781
Antidiarrheal drugs	1.106 (0.994–1.218)	0.051	0.379
Antithrombotic drugs	1.073 (0.955–1.197)	0.224	0.781
Corticosteroids for systemic use	1.043 (0.952–1.133)	0.348	0.819
Antibacterial drugs for systemic use	1.083 (0.990–1.174)	0.067	0.425
Antimycotic drugs for systemic use	1.035 (0.944–1.126)	0.454	0.819
Antimicrobial drugs for mycobacterial infections	1.089 (1.023–1.155)	0.006	0.063
Anti-inflammatory drugs	1.070 (0.970–1.170)	0.158	0.700
Antiprotozoal drugs	1.037 (0.953–1.123)	0.394	0.819
Anthelminthic drugs	1.068 (1.008–1.127)	0.021	0.189

Our findings suggest that the increase in CA-CDI proportion was not associated with population-level medication exposure in Queensland during 2008–2012. CA-CDI epidemiology in Queensland might be driven by a group of factors other than medication exposure, such as transmission of the pathogen from food, animals, or hospitals into the community. Studies have confirmed the risk for foodborne and animalborne spread of *C. difficile* into the community (*3*). In Australia and New Zealand, importation of onions and garlic from the United States and Mexico might be responsible for increased CDI cases during Southern Hemisphere summers (*4*), and high prevalence of *C. difficile* colonization in piglets has been identified (*5*). However, the role of these factors in leading to CA-CDI cases remains unknown.

A recent contact tracing study in the United Kingdom demonstrated that a considerable proportion of CDIs among patients in healthcare settings originated from the community (*6*); this finding was supported by another study, which showed that in Queensland, more than two thirds of patients with CA-CDI required hospitalization (*7*). Currently, there is no evidence of a reverse-infection route (healthcare-acquired CDI being transmitted to persons in the community). However, Sethi et al. documented environmental shedding of *C. difficile* by inpatients for several weeks after resolution of symptoms (*8*). Therefore, the possibility that asymptomatic patients might be a source of transmission after hospital discharge needs to be examined. In recent years, epidemiologic models exploring the role of CDI coming from the community into the hospital have become increasingly popular (*9*); however, to the best of our knowledge, only 1 modeling study described CDI dynamics within the wider community (*10*). Although this approach is innovative, we acknowledge some limitations. Medication exposure was used as a proxy, based on the average prescription in the community, and it cannot be applied to the individual patient. In addition, we were unable to adjust the regression model for the presence of concurrent medical conditions and other unmeasured confounders.

Exposure to medications, particularly antimicrobial drugs, probably influences CA-CDI pathogenesis (*2*). However, our community-based assessment indicates that a more holistic exploration is needed to identify alternative factors driving increases in CA-CDI cases in the wider population.
